# An Investigation of an Acute Gastroenteritis Outbreak: *Cronobacter sakazakii*, a Potential Cause of Food-Borne Illness

**DOI:** 10.3389/fmicb.2018.02549

**Published:** 2018-10-26

**Authors:** Wei Yong, Baofu Guo, Xiaochao Shi, Tingting Cheng, Mingming Chen, Xiao Jiang, Yanhua Ye, Junning Wang, Guoxiang Xie, Jie Ding

**Affiliations:** ^1^Microbiology Laboratory, Nanjing Municipal Center for Disease Control and Prevention, Nanjing, China; ^2^Department for Nutrition and Food Hygiene, Nanjing Municipal Center for Disease Control and Prevention, Nanjing, China; ^3^Microbiology Laboratory, Jiangning District Center for Disease Control and Prevention, Nanjing, China; ^4^Zeta Biosciences (Shanghai) Co., Ltd., Shanghai, China

**Keywords:** *Cronobacter sakazakii*, gastroenteritis, food-borne illness, whole genome sequencing, single nucleotide polymorphism, PFGE, MLST

## Abstract

Whole genome sequencing (WGS) has been widely used in traceability of food-borne outbreaks nowadays. Here, an interesting connection between *Cronobacter sakazakii* and food-borne acute gastroenteritis (AGE) was noticed. In October 2016, an AGE outbreak affecting 156 cases occurred in a local senior high school. Case-control study including 70 case-patients and 295 controls indicated a strong association between eating supper at school canteen of the outbreak onset and AGE, as revealed by the Odds Ratio (OR: 95.32). Six recovered *Cronobacter* strains were evaluated and compared using pulsed-field gel electrophoresis (PFGE), multilocus sequence typing (MLST) and WGS. A phylogenetic tree of whole genomic single nucleotide polymorphisms (wgSNPs) were generated to traceback the potential contamination source in this outbreak. *C. sakazakii* isolates S2 from a patient’s rectal swab and S4 from leftover food sample shared identical PFGE pattern and sequence type (ST73), and clustered tightly together in the SNP phylogenetic tree. *C. sakazakii* isolates S5 and S6 from food delivery containers were both ST4 but with different PFGE patterns. *Cronobacter* isolates S1 and S3 from two patients’ rectal swab were sequenced to be *C. malonaticus* and shared another PFGE pattern (ST567). The interesting feature of this study was the implication of *C. sakazakii* as a causative agent in food-borne AGE occurring in healthy adults, although *C. sakazakii* is considered as an opportunistic pathogen and generally affects neonates, infants and immunocompromised adults.

## Introduction

*Cronobacter* spp. (formerly defined as *Enterobacter sakazakii*) is a member of the family Enterobacteriaceae, and is known as emerging opportunistic food-borne pathogen for humans. Most of the *Cronobacter* infections were reported to attack neonates, infants, and adults with immunocompromised condition (See et al., 2007; Healy et al., 2010). The consumption of powdered infant formula (PIF) has been epidemiologically implicated in food-borne illness outbreaks among infants which were linked with *Cronobacter* spp., of which *C. sakazakii* and *C. malonaticus* were the two species that most commonly detected (van Acker et al., 2001; Cai et al., 2013; Fei et al., 2015). Moreover, *Cronobacter* spp. was reported to have the capacity to cause nosocomial outbreaks in neonatal intensive care unit (NICU) in different countries (van Acker et al., 2001; Caubilla-Barron et al., 2007; Hariri et al., 2013). This organism is widely distributed in nature and has been isolated from various environmental sources including PIF production facilities, hospitals, and households (Masaki et al., 2001; Kandhai et al., 2004; Mullane et al., 2007), and from different food categories such as dried food, produce, spice, and even expressed breast milk (Baumgartner et al., 2009; Beuchat et al., 2009; Healy et al., 2010; McMullan et al., 2018). Xu has reported that 18.6% out of 280 ready-to-eat retailer foods were positive for *Cronobacter* genus (Xu et al., 2015). Liu reported that the positive rate of *Cronobacter* spp. and *C. sakazakii* was 0.54–0.27% in healthy adults, respectively, slightly lower than 1.2–0.58% in adults with acute diarrheal illness, respectively, and with no statistically difference (Liu et al., 2013).

In October 2016, an outbreak of AGE affecting 156 cases occurred in a local senior high school, and the patients involved in this outbreak showed mild clinical symptoms and generally recovered within 2 days without hospitalization and severe cases. No common enteric pathogens were identified from the collected 105 samples including rectal swabs, leftover foods and environmental swabs. Meanwhile, the laboratory investigation identified 31 samples which were positive for *E. sakazakii* (*Cronobacter* spp.) nucleic acid and isolated six *Cronobacter* strains. The case-patients were more likely than the healthy employees to carry *Cronobacter* spp., and *Cronobacter* spp. was only detected in the supper which was epidemiologically linked with the outbreak. As *Cronobacter* spp. is not commonly regarded as a causative agent for AGE in healthy adults, we further conducted genetic characterization of the six strains including PFGE, MLST, and whole genome SNP phylogeny to track infection source and give more laboratory evidence.

Thus far, WGS has been widely used as a diagnostic tool to improve the level of resolution in investigating outbreaks (Kan et al., 2018). In this study, the two *C. sakazakii* strains isolated from a potentially contaminated food and a patient’s rectal swab separately were nearly identical with only 5 SNPs differences as determined by the wgSNP phylogenetic analysis, which could be viewed as supportive evidence for *C. sakazakii* being the etiological agent. Combined with other laboratory results and epidemiological analysis which indicated the possible link between *C. sakazakii* and the outbreak, we strongly suggested that *C. sakazakii* may lead to intestinal disorders independently and even cause AGE outbreaks in some occasions.

## Materials and Methods

### Case Definition and Case Finding

At the time the suspected food-borne illness outbreak was reported, Jiangning District Center for Disease Control and Prevention conducted field epidemiology investigation at a local senior high school where the outbreak occurred. Suspected cases were defined as those with an onset of three or more episodes of loose stools, or one or more episodes of vomiting within a 24 h period. Probable cases were suspected cases with clinical diagnosis and treatments. Confirmed cases were suspected or probable cases with positive etiological agent. Individual case investigation was conducted and epidemiological data were collected to know the symptoms, date of onset, duration of illness, clinical treatment, and food exposure data assuming an incubation period of 4–24 h.

### Case-Control Study

Suspected or probable or confirmed cases were randomly selected across the three grades. The control students were selected randomly from asymptomatic students in the class or the neighboring class where the symptom cases were reported. Altogether 70 cases and 295 controls were enrolled in the study. Data about activities of case individuals and controls were collected and analyzed.

### Sample Collection and Preliminary Laboratory Examination

A total of 105 specimens were collected, including 35 leftover food samples, 10 swabs from the catering company’s processing environment, 27 rectal swabs from patients, 20 rectal swabs from employees in the school canteen and the catering company and 13 from food delivery carts and boxes. All aspects of the study were in accordance with national ethics regulations and approved by Science and Technology Committee of Nanjing Municipal Center for Disease Control and Prevention. Since the data obtained in the present study were de-identified, the requirement for written consent from the patients was waived.

Total nucleic acids of these specimens were extracted (Shanghai Fosun Long March Medical Science, Shanghai, China) for real-time fluorescence PCR assays to identify the presence of common food-borne pathogens using a panel of PCR detection kits (Beijing Applied Biological Technologies, Beijing, China), including *Salmonella* spp., *Shigella* spp., *Vibrio* spp., enteropathogenic *Escherichia coli, Staphylococcus aureus, Bacillus cereus, E. sakazakii, Campylobacter jejuni, Yersinia enterocolitica*, and *Listeria monocytogenes*, as well as norovirus, rotavirus, astrovirus, adenovirus, and sapovirus. The real-time PCR kit for the detection of *E. sakazakii* used bacterial outer membrane protein A (ompA) gene as the target and the reaction was performed on ABI 7500 fast real-time PCR platform (Thermo Fisher Scientific, Rockford, IL, United States). The amplification conditions were set following the manufacturer’s instructions. Meanwhile, according to the standardized isolation protocols suggested in Food-borne Disease Surveillance Manual by China National Center for Food Safety Risk Assessment and National Food Safety Standards series, routine bacterial culture was performed for all the specimens.

### Bacterial Isolates and PFGE

Six isolates of *Cronobacter* spp. were obtained from the routine bacterial culture process including three from patient rectal swabs, one from a dish called knotted thin sheets of bean curd with braised pork, and two from the swabs of food delivery boxes. Briefly, rectal swabs, environmental swabs, and pre-enriched and enriched food samples were plated onto *E. sakazakii* Chromogenic Medium (Land Bridge, Beijing, China) and incubated at 36°C for 24 h to isolate *Cronobacter* strains. Presumptive *Cronobacter* colonies were confirmed by ATB Expression and ID32E cards for the identification of *Enterobacteriaceae* and other non-fastidious Gram-negative rods (bioMerieux, Marcy L’Etoile, France). PFGE was then performed according to the standard PulseNet protocol^1^, using the restriction enzyme Spe1 (TaKaRa Clontech, Kusatsu, Shiga, Japan). Gels were run in 1% agarose gels with a CHEF-DRIII system (Bio-Rad, Hercules, CA, United States). The electrophoretic patterns were visualized by UV illumination, and analyzed using BioNumerics software version 7.5 (Applied Maths, Kortrijk, Belgium). Banding patterns were analyzed by using the Dice similarity coefficient, and a dendrogram was generated by UPGMA algorithm (unweighted pair-group method with arithmetic averages), with a setting of 1.5% for optimization and position tolerance of 1.5%.

#### Antibiotic Sensitivity Testing

The antibiotic susceptibility of the six recovered *Cronobacter* isolates was tested using Gram Negative MIC plates GN4F (Thermo scientific, MA, United States) against fifteen antibiotics, including ampicillin (AMP), ampicillin/sulbactam (AMS), cefotaxime (CTX), ceftazidime (CAZ), trimethoprim/sulfamethoxazole (SXT), imipenem (IPM), tetracycline (TET), cefazolin (CFZ), cefoxitin (CFX), gentamicin (GEN), chloramphenicol (CHL), ciprofloxacin (CIP), erythrocin (ERY), nalidixic acid (NAL), and azithromycin (AZM). After incubation at 36°C for 18 h, the plates were read and interpreted automatically by Sensititre Vizion Digital MIC Viewing System. The results were manually checked according to the Manual of Molecular Typing and Antibiotic Susceptibility Test of Food-borne Pathogenic Bacteria by Jiangsu Province, and then were nominated as sensitive (S), resistant (R) and intermediate resistant (IR).

### DNA Preparation, Whole Genome Sequencing and Assembly

Genomic DNA of each isolates was extracted from overnight cultures using the E.Z.N.A^®^ Bacterial DNA Kit (Omega Bio-tek, Norcross, GA, United States). A 500 bp library of these isolates was constructed and whole genome sequencing (WGS) was performed using Illumina Miseq platform (Illumina Inc., San Diego, CA, United States) with a 250 bp paired-end read according to the manufacturer’s instructions at approximate 100 × coverage. Genomic sequence contigs were *de novo* assembled using the CLC Genomics Workbench ver 9.0 (Qiagen, Duesseldorf, Germany) and MicrobeTrakr Plus (Zeta Biosciences, Shanghai, China). Quake (Kelley et al., 2010) and BWA (Li, 2013) were used in pre- and post-assembly sequences correction, respectively.

### *In silico* MLST Analysis

The initial analysis and identification of the isolates were performed using an *in silico* *Cronobacter* spp. MLST approach, based on the information available at the *Cronobacter* spp. MLST website^2^. Seven *Cronobacter* spp. loci (*atp*D, *fus*A, *gln*S, *glt*B, *gyr*B, *inf*B, and *pps*) previously described (Baldwin et al., 2009) were used for MLST analysis. The same *Cronobacter* spp. MLST database was also used to assign numbers for alleles and STs.

### SNP Calling and Phylogenetic Analysis

A total of 158 *C. sakazakii* assemblies of a global collection were obtained from NCBI Assembly Database. By mapping onto the reference genome *C. sakazakii* strain ATCC 29544 (GenBank assembly accession number GCA_000982825.1) and enabling the recombination filter, whole genomic single nucleotide polymorphisms (wgSNPs) of all the retrieved *C. sakazakii* assemblies together with our isolates were identified. The maximum likelihood phylogeny of the wgSNPs was determined by the software package Harvest Suite (version 1.2) (Treangen et al., 2014). The Harvest was run in default configuration which was optimized for the closely related bacterial genomes analyzed in this study.

### Nucleotide Sequence Accession Number

The accession numbers of the six *Cronobacter* isolates in NCBI database were PQJL00000000, PQJM00000000, PSON00000000, PQJN00000000, PQJO00000000, and PQJP00000000.

## Results

### Epidemiologic Analysis

**FIGURE 1:**
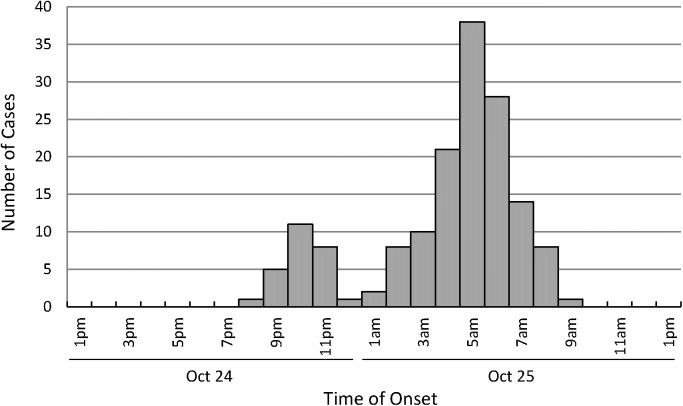
Onset of the acute gastroenteritis outbreak from October 24–25, 2016.

There were a total of 1656 students in 39 classes across three grades in the local high school where the survey was conducted. On the day of outbreak onset, lunch and dinner were both provided by a catering company outside school with the meal cooked, box-packed and delivered to school and then distributed by employees in the school canteen. According to the case definition, 156 symptomatic individuals were preliminarily identified, including 124 suspected cases, 12 probable cases, and 20 confirmed cases. All the suspected cases were students except two members of school staff. The symptoms were generally mild. Most of the cases experienced diarrhea (99.4%) and abdominal pain (81.4%), a proportion of individuals presented nausea (16.7%), and a few vomited (3.8%). The index case occurred on the evening of 24th October at 8 pm. A small peak on 24th October at 10 pm and a main peak on 25th October at 5 am were depicted on the epidemic curve. The duration between the first and the last case was 14 h (Figure 1). The epidemic curve suggested that it was a point source outbreak.

Sixty-seven of 70 cases (95.7%) and 56 of 295 controls (19.0%) ate supper at school on the evening of 24th October (Odds Ratio (OR):95.32; 95% confidence interval (CI):28.92–314.15). The stratified analysis further identified eating supper at school on the evening of 24th October as the only activity with a statistically significant OR.

### Real-time PCR Analysis, Bacterial Isolation, and Molecular Typing

31 samples were positive for nucleic acid of *Cronobacter* spp. using real-time PCR, including 20 patients’ rectal swabs, 3 employees’ rectal swabs, 4 leftover food samples of the supper on 24th October and 4 environmental swabs from food delivery containers. After enrichment and bacterial isolation culture, six isolates of *Cronobacter* spp. were obtained including three from patient rectal swabs, one from a leftover food sample of the supper on 24th October, the dish of knotted thin sheets of bean curd with braised pork, and two from the environmental swabs of food delivery boxes. None of other common food-borne bacteria and gastroenteritis-causing viruses mentioned in the Materials and Methods section was detected.

No *Cronobacter* spp. isolates was recovered from 20 healthy employees in the school canteen and the catering company. In contrast, 3 *Cronobacter* spp. strains were isolated from 27 patients and the positive rate was much higher and reached 11.1%. The difference between the two sampling groups was statistically significant (*p* < 0.05). With respect to the PCR results, the positive rate of *Cronobacter* spp. nucleic acid for the two groups was 15.5% (3/20) and 74.1% (20/27), respectively, with a statistically significant difference (*p* < 0.05). Besides, the positive rate of *Cronobacter* spp. nucleic acid for lunch and supper for the school was 0 and 40%, respectively (*p* < 0.05).

**FIGURE 2:**
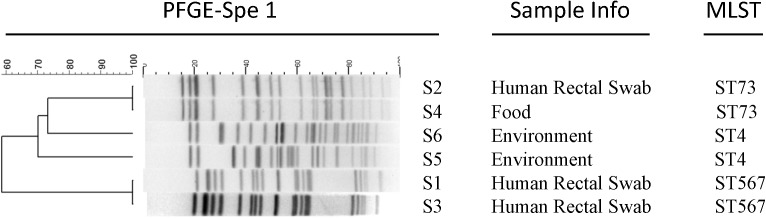
PFGE and MLST analysis of the six *Cronobacter* isolates in this outbreak.

Pulsed-field gel electrophoresis was subsequently carried out for these six recovered *Cronobacter* spp. isolates (Figure 2). Isolates S2 from a patient’s rectal swab and S4 from the leftover food sample exhibited identical PFGE pattern. Isolates S1 and S3, from two patients’ swabs separately, showed another same PFGE pattern. Isolate S5 and S6, both from swabs of food delivery boxes, exhibited different PFGE patterns.

*In silico* MLST analysis was performed straightly on the WGS assemblies. Consistent with the PFGE results, isolates S2 and S4 both belonged to sequence type (ST) 73. Isolates S5 together with S6 were both grouped to ST4. Isolate S1 and S3 both belonged to a newly identified sequence type 567.

### Comparative Phylogenetic Genome Analysis

**FIGURE 3:**
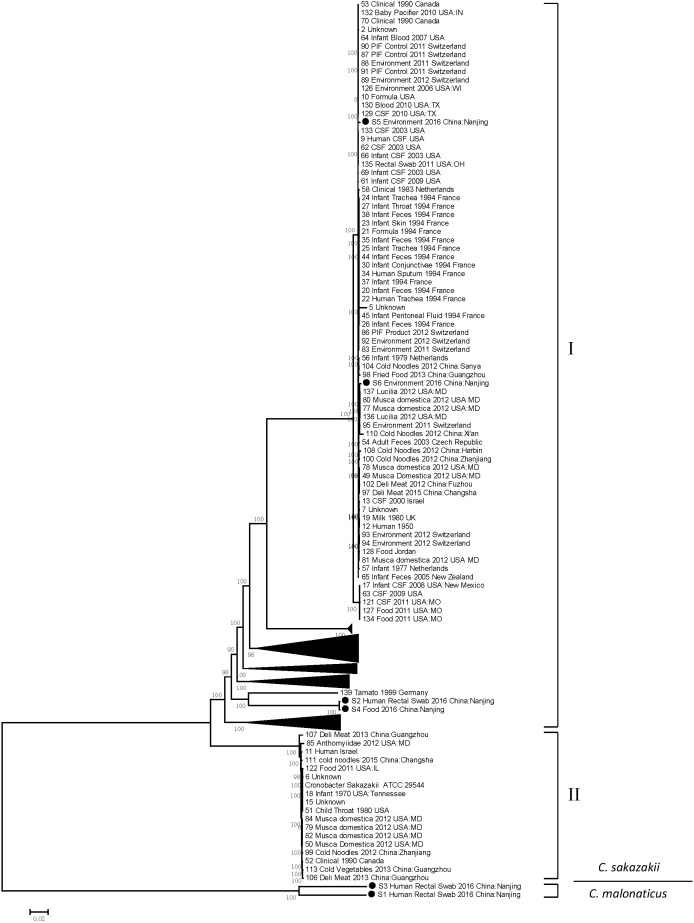
Maximum likelihood phylogenetic tree based on single nucleotide polymorphism (SNP) analysis of the present six *Cronobacter* isolates and comparison sequences. Black solid circles (∙) indicate the *Cronobacter* isolates in this study. Numbers along branches are bootstrap values. Scale bar indicates estimated evolutionary distance.

After sequencing and assembly (detail in Supplementary Table 1), the six *Cronobacter* strains came out to be two species: *C. sakazakii* and *C. malonaticus*. Thereinto isolates S2, S4, S5, and S6 were *C. sakazakii* strains, and S1 together with S3 were *C. malonaticus* strains. Whole genome SNPs extracted from WGS data of present isolates presented were subjected to comparative phylogenetic genome analysis and generated a comprehensive phylogenetic tree (Figure 3). The *C. sakazakii* strains can be partitioned into two distinct lineages separated by >25000 SNPs. Within each lineage, only hundreds, at most thousands of unique SNPs existed. Lineage I comprised 145 strains including our present isolate S2, S4, S5, and S6. *C. sakazakii* isolates S2 from a patient rectal swab and S4 from a leftover food sample formed a tight cluster with a distance of only five SNPs, which showed a critical epidemiological clue and suggested a possible transmission route. Isolates S5 and S6 from two food delivery boxes scattered in another cluster and 149 SNPs were observed between them. No present isolates appeared in lineage II. The two *C. malonaticus* strains S1 and S3 exhibited a distance of 312 SNPs.

#### Antibiotic Susceptibility Analysis

The antibiotic susceptibility profile of the 6 isolated *Cronobacter* strains to 15 different antibiotics (Table 1) revealed that all the 6 isolates were resistant or intermediate resistant to CFZ and ERY (100%). Two strains isolated from environmental samples, S5 and S6, were resistant to AMP (33.3%). It was also found that all the six *Cronobacter* strains were sensitive to AMP/AMS, CTX, CAZ, SXT, IPM, TET, CFX, GEN, CHL, CIP, NAL, and AZM.

**Table 1:** Antibiotic sensitivity and resistance profile of the six *Cronobacter* isolates.

	AMS	CTX	CAZ	SXT	IPM	TET	CFZ	CFX	GEN	AMP	CHL	CIP	ERY	NAL	AZM
SI	<=	<=	<=	<=	<=	<=			<=	<=	<=	<=			<=
	2	0.25	1	0.25	0.25	1	4	4	1	2	2	0.03	32	4	4
	S	S	S	S	S	S	IR	S	S	S	S	S	R	S	S
S2	<=	<=	<=	<=	<=	<=			<=	<=		<=		<=	<=
	2	0.25	1	0.25	0.25	1	4	4	1	2	4	0.03	64	2	4
	S	S	S	S	S	S	IR	S	S	S	S	S	R	S	S
S3	<=	<=	<=	<=	<=	<=			<=	<=	<=	<=		<=	<=
	2	0.25	1	0.25	0.25	1	4	4	1	2	2	0.03	16	2	4
	S	S	S	S	S	S	IR	S	S	S	S	S	R	S	S
S4	<=	<=	<=	<=	<=	<=			<=	<=		<=		<=	<=
	2	0.25	1	0.25	0.25	1	4	4	1	2	4	0.03	32	2	4
	S	S	S	S	S	S	IR	S	S	S	S	S	R	S	S
S5	<=	<=	<=	<=	<=	<=		<=	<=		<=	<=		<=	<=
	2	0.25	1	0.25	0.25	1	8	2	1	16	2	0.03	16	2	4
	S	S	S	S	S	S	R	S	S	IR	S	S	R	S	S
S6	<=	<=	<=	<=	<=	<=			<=			<=		<=	<=
	2	0.25	1	0.25	0.25	1	8	8	1	16	4	0.03	64	2	4
	S	S	S	S	S	S	R	S	S	IR	S	S	R	S	S
Isolates resistance % (IR+R)	0	0	0	0	0	0	100	0	0	33.3	0	0	100	0	0

## Discussion

This outbreak was unusual in that it was assumed to be caused by *Cronobacter* spp., an opportunistic organism that is usually not considered to cause acute gastroenteritis in healthy individuals, and *C. sakazakii* here showed a strong link between contaminated food and an AGE patient. Although the epidemiology of *Cronobacter* spp. has not been well elucidated, it was revealed that *Cronobacter* spp. has been linked to PIF contamination and thus given rise to a number of food-borne illness outbreaks occurring in neonates and infants. Lai summarized the clinical relevance of this organism based on literature review and case reports in the author’s institution (Lai, 2001). *Cronobacter* spp. often caused meningitis (or concomitant bacteremia) among neonates and infants, and urinary tract infection and hemorrhagic diarrhea were also observed in some occasions. *Cronobacter* spp. caused infections in adults with serious underlying health issues as well, and developed to invasive diseases such as bacteremia, urosepsis, osteomyelitis, and pneumonia (Hawkins et al., 1991; See et al., 2007).

Although the reports of *C. sakazakii* infection in healthy adults are rare, in this case, there were reasonable facts that supported *C. Sakazakii* to be a possible causing agent. First, *Cronobacter* spp. strains have been reported to exhibit capacity to adhere to and enter intestinal cells *in vitro* (Quintero-Villegas et al., 2014; Ye et al., 2016). The production of enterotoxin and the presence of bacterial ompA and lipopolysaccharide (LPS) play a role in the host–pathogen interactions (Ye et al., 2016). Second, although we identified two different species of *Cronobacter* spp. in the patients’ samples, the *C. sakazakii* strains S2 from one patient’s rectal swab sample and S4 from a potential contamination food formed a tightly clustered group with only five SNPs identified using the high-level-resolution WGS technique. The dish of knotted thin sheets of bean curd with braised pork, from which the *C. sakazakii* strain S4 was identified, was only served in the supper on 24th October which has been demonstrated by case-control study to be strongly related to the outbreak. In addition, based on PCR results, 4 out of 10 food samples from the supper of 24th October were found to harbor *Cronobacter* spp., while 0 out of 10 sample was positive for *Cronobacter* spp. from the lunch on the same day. Third, at least in this outbreak, *Cronobacter* spp. seemed more likely to colonize case-patients’ than healthy employees’ intestinal tract, as shown by the real-time PCR and bacterial isolation results for *Cronobacter* genus. Combined with the fact that *Cronobacter* spp. affects neonates more seriously than adults and colonizes more likely in neonate’s intestinal tract, it can be inferred that there might be an association between the carriage of *Cronobacter* spp. in the intestinal tract and clinical outcomes. The frequency of this organism being recovered from a specific population’s intestinal tract might in some degree correlate whether the target individuals are sick or not. Last but not least, the symptoms presented in this outbreak were generally mild and had a short duration, which was consistent with the clinical manifestations caused by opportunistic organisms in healthy adults.

The limitation of this investigation was that the recovered *Cronobacter* strains were not sufficient enough and there were two species of *Cronobacter* detected in this outbreak. Had we recovered more *Cronobacter* strains from the samples, we should have gathered more evidence to support our conclusion that *C. sakazakii* was the cause of the outbreak. The overall isolation rate out of 31 PCR positive samples was 19.3% (6/31) in this study. The low bacterial recovery can be explained as follows: firstly, real-time quantitative PCR is much more sensitive than culture-based methods (Law et al., 2014); secondly, pathogen type (Imdad et al., 2018), bacterial load, and the status and viability of the bacteria all affect the results; thirdly, we used rectal swabs instead of stool samples for *Cronobacter* isolation, which may suggest the importance of sampling strategies for this organism. Based on the subtyping of the 6 *Cronobacter* isolates, we assumed that the two *C. malonaticus* strains from two patients were unrelated to the outbreak, as the distance of 312 SNPs was ambiguous to illustrate the epidemiological relationship, whereas the distance of 5 SNPs between the two *C. sakazakii* strains confirmed their close correlation and link the patient to the leftover food. In food-borne illness outbreaks, the existence of variable *Cronobacter* species in humans and environment/food makes source tracking difficult, only high-resolution methods like wgSNP phylogeny could successfully distinguish outbreak strains from non-outbreak strains. We nevertheless could not rule out the possibility that *C. malonaticus* may also contribute to the outbreak. If we had got more *C. malonaticus* strains and supportive molecular evidences, we would suggest that *Cronobacter* spp. cause the outbreak.

The pathogenicity and epidemiology of *Cronobacter* spp. have not been well described, which led to the difficulty in the prevention, treatment, and control of this organism. Opportunistic pathogens like *Proteus* spp. and *Clostridium perfringens*, which pose a threat of poisoning when the contaminated foods are consumed, have been included in diagnostic criteria for food poisoning events. Given the fact that *Cronobacter* spp. might contribute to food-borne enteric infections in healthy adults and that this organism shows high resistance to disadvantageous environmental conditions, relevant criteria of this organism regarding the bacterial load limitation in food items other than PIF should be considered when necessary.

## Conclusion

In conclusion, based on epidemiological evidence and the laboratory results, including the application of WGS to improve the differentiation of bacterial isolates, we suggest that *C. Sakazakii* poses a previously underestimated threat on food safety, as *C. Sakazakii* has not been universally implicated as the causing agent of food-borne illness outbreaks occurring in healthy adults. Studies of future food-borne illness outbreaks should consider exposures to *C. Sakazakii*, when the positive rate of this organism was higher than average in suspected food supply, or in the individuals suffering from diarrhea. The accumulation of the molecular epidemiological information on *C. Sakazakii* would contribute to better understanding this organism and setting related food safety criteria.

## Author Contributions

JD designed the study and revised the article. WY coordinated the study, carried out the experiments of whole genome sequencing and assembly, phylogenetic analysis, and wrote the manuscript. BG did the epidemiological investigation and analysis. XS, TC, MC, XJ, and YY performed the PCR for pathogen screening, bacterial isolation, PFGE, and MLST. JW did the SNP calling and phylogenetic analysis. GX gave the comprehensive guidance of experiments.

## Conflict of Interest Statement

The authors declare that the research was conducted in the absence of any commercial or financial relationships that could be construed as a potential conflict of interest.

## Footnotes

1 http://www.cdc.gov/pulsenet/pathogens/index.html

2 https://pubmlst.org/cronobacter/
