# Supplementary material for: An Investigation of an Acute Gastroenteritis Outbreak: Cronobacter sakazakii, a Potential Cause of Food-Borne Illness
Source: Front Microbiol. 2018 Oct 26;9:2549. doi: 10.3389/fmicb.2018.02549 (PMC6214019; doi:10.3389/fmicb.2018.02549)
Supplement: Supplementary file 1 [file Table_1.DOCX]

Supplementary Table 1. Total raw reads, GC content, N50 value of WGS assembly and Average contig length.

|  | Total Reads | GC content | N50 | Average contig length |
| --- | --- | --- | --- | --- |
| S1 | 2,254,472 | 57% | 548,953 | 16554 |
| S2 | 7,805,360 | 56.8% | 555,072 | 30224 |
| S3 | 3,767,678 | 56.9% | 75,553 | 12252 |
| S4 | 3,185,052 | 56.7% | 345,787 | 63501 |
| S5 | 784,004 | 56.7% | 78,539 | 23601 |
| S6 | 457,818 | 56.6% | 17,785 | 7355 |
